# QFASA: A Comprehensive R Package for Diet Estimation via Fatty Acid Signature Analysis

**DOI:** 10.1002/ece3.71090

**Published:** 2025-03-12

**Authors:** Connie Stewart, Justin Kamerman, Jennifer McNichol, Holly Steeves, Tyler Rideout

**Affiliations:** ^1^ University of New Brunswick Saint John New Brunswick Canada; ^2^ Instnt Inc. New York New York USA; ^3^ Simon Fraser University Burnaby British Columbia Canada; ^4^ Western University London Ontario Canada

**Keywords:** calibration coefficient estimation, diet estimation, fatty acids, QFASA, software

## Abstract

Quantitative fatty acid signature analysis (QFASA) is a well‐established diet estimation method that has been used extensively on a wide variety of marine mammal species. The method, along with its new refinements and extensions, requires the use of statistically intricate tools, many of which are computationally demanding. Recent developments in QFASA include a maximum likelihood framework for diet estimation, statistically valid inference procedures such as confidence intervals for the diet and hypothesis tests for comparing fatty acid signatures and/or diets, a measure of repeatability in the diet estimates, a prey species selection algorithm, as well as novel ways to estimate calibration coefficients, which are used to improve accuracy in the estimates. The QFASA R package was developed to facilitate access to the latest statistical QFASA tools and provide a means of efficiently disseminating new QFASA‐related research, often developed by statisticians in collaboration with biologists. Further, using up‐to‐date functions ensures that QFASA methods are being applied in a legitimate and consistent manner. In this work, we present the QFASA R package, highlighting key functions for diet estimation and demonstrating their use with sample data available in the package. The QFASA R package is user‐friendly, offers a broad range of functionality, and the vast majority of the functions are unique to this package.

## Graphical abstract

Quantitative fatty acid signature analysis (QFASA) is a well‐established diet estimation method that has been used extensively on a wide variety of marine mammal species. The method requires the use of statistically intricate tools, many of which are computationally demanding. The QFASA R package allows ecologists to access the latest statistical QFASA methodology and provides a means of efficiently disseminating new related research.

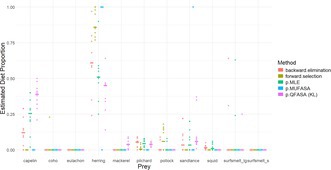

## 1 Introduction

Quantitative fatty acid signature analysis (QFASA) (Iverson et al. 2004) has played a key role in understanding predator–prey relationships since its conception nearly two decades ago. In general terms, QFASA relies on the knowledge that some fatty acids (FAs) of prey are, in essence, directly deposited in the lipid storage sites of a predator. In the first generation of QFASA, estimates of the proportion of each of several potential prey in the predator's diet were obtained by minimizing the distance between the predator's FA signature and average prey signatures (one for each prey type) using statistical techniques. QFASA has been successfully applied to a variety of animals including seals (Tucker et al. 2009), fishes (Happel et al. 2016), polar bears (Rode et al. 2014; Stern et al. 2024), seabirds (Wang et al. 2010; Wang et al. 2014), orcas (Remili et al. 2023), and dolphins (Xie et al. 2022). Compared to other indirect diet estimation methods, QFASA has the advantage of being non‐lethal and providing diet estimates reflective of a predator's longer‐term diet (Budge et al. 2006).

Calibration coefficients (CCs) and fat content are two important components of the QFASA model. CCs are assumed constants that account for the metabolism process that may result in some FAs in the predator being higher or lower than in the corresponding prey FAs. For most predators, CCs improve accuracy and are a necessary part of the model but are unknown in practice. Through captive studies, CCs have been estimated for a variety of species (Zhang et al. 2020). Adjusting for the fat content of the prey involves a re‐scaling of the final diet estimate, resulting in the estimated proportions of biomass being converted to the estimated proportion of the number of animals consumed. The fat content or percentage lipid is determined by biologists when the prey is sampled and analyzed.

Initial code to carry out the basic QFASA algorithms was developed by Iverson et al. (2004) and, originally, was shared to QFASA users on an individual basis as needed. A more detailed history of QFASA scripts may be found in (Bromaghin 2017). As QFASA grew in popularity and began to evolve with the development of extensions and new accompanying inference methodology, it became worthwhile and necessary, from a practical point of view, to develop a QFASA R package. The first version of the R package “QFASA” was released on CRAN in May, 2016. While this version contained only basic functionality for QFASA diet estimation, along with a few additional refinements and options, a more comprehensive version is now available. A complementary package called qfasar (Bromaghin 2017) was made available on CRAN shortly after the QFASA package was released (August, 2016) and is described as providing functions for “data preparation; diet estimation; diagnostics and simulation support”. Although the QFASA and qfasar packages contain some overlapping functionality (e.g., both contain functions to compute the original QFASA diet estimates and functions for simulation and diagnostics), the QFASA R package encompasses the substantial recent methodology that has been developed to address shortcomings in the QFASA procedure. An overview of the package and its functionality is discussed in materials and methods, a dedicated section on examples provides results using the sample data in the package, and the last section on discussion outlines concluding remarks.

## 2 Materials and Methods

The core functions in the QFASA R package can be grouped according to their functionality as follows: Diet Estimation, Inference, Repeatability, and Model Assessment.

### 2.1 Diet Estimation

Given predator and prey FA signatures, a variety of methods have been implemented in the QFASA package that attempt to match the predator and prey signatures to yield diet estimates. Several of these methods require experimentally derived CCs, and these are treated as known constants. Recently, methods that simultaneously estimate both the diet estimates and the CCs have been proposed. The package's diet estimation functions, categorized by whether CCs are assumed known or estimated, are described below.

#### 2.1.1 CCs (Assumed) Known

QFASA is carried out in the package using the function p.QFASA. The function p.QFASA allows diet estimates to be calculated via three choices for measuring distance, namely the Aitchison (AIT) distance (Stewart and Field 2011), Kullback–Leibler (KL) distance (Iverson et al. 2004) or the chi‐square (CS) distance (Stewart 2016). The diet estimate corresponding to each row in the predator matrix is computed, along with the modeled FA signature (a linear combination of the prey mean FA signature and the estimated diet proportions), the contribution of each FA to the final minimized distance, as well as the final minimized distance between each predator and the modeled predator FA signature.

The function p.QFASA requires as input a set of CCs, determined from experimental studies, and transforms the predator FA signatures to the prey space prior to computing the diet estimates. If no CCs are provided, a vector of ones is used. Fat content is assumed to be a vector of ones (i.e., the default is no re‐scaling of the diet estimates), unless a vector of mean fat content for each prey type is supplied. A vignette on modeling workflow for this function is provided with the package: QFASA Workflow Example (r‐project.org) to assist with setting up the function inputs and extracting the outputs.

The QFASA package offers the option of obtaining estimates derived from the new maximum likelihood estimation (MLE) framework (Steeves 2020) referred to as “Maximum Unified Fatty Acid Signature Analysis”, or MUFASA, using the function p.MUFASA. The model assumes unobserved random effects in place of the prey type sample means (to account for the predator not consuming prey in the sampled prey database) and an error term. The likelihood function to be optimized assumes that the log‐ratio transformed data are multivariate normal. While estimates obtained through this function have nice statistical properties, this function is computationally intensive as numerical integration is required to integrate out random effects and this is carried out in C++ using the Template Model Builder (TMB) package. For this function, the modeling workflow vignette is also provided: MUFASA Workflow Example (r‐project.org).

A new approach to FA diet estimation has recently been proposed, involving the use of stepwise selection algorithms to select an optimal subset of prey types as determined by the minimization of a chosen information criterion (IC) (Rideout n.d.). Both forward selection and backward elimination methods have been developed, and the associated functions forward.selection and backward.elimination return final diet estimates computed with the selected subset of prey types, along with other relevant information about the selection process. The prey selection algorithm forward.selection is particularly useful when the prey database contains a large number of prey types since, when the number of prey types exceeds the number of FAs, the QFASA diet estimates may not be unique. The default argument *k* = 2 corresponds to a choice of the Akaike information criterion (AIC) for selection. Unique to the backward elimination method, the default argument of cutoff = 0.1 ensures that species with a proportion of 0.1 or greater in the estimated diet of any individual predator will not be considered for removal from the prey set. In the case of forward selection, the default argument min.spec = 5 means that at least five prey types will be included in the selected prey set. The model and corresponding likelihood used for deriving the estimates are similar to those underlying the p.MUFASA model but simplified by the replacement of random effects with the sample means of prey species for quicker computation. The function p.MLE returns diet estimates based on this simpler MLE model, which, in the case of constant variance among the FAs, can be shown (Rideout n.d.) to be equivalent to p.QFASA using the AIT distance.

#### 2.1.2 CCs Estimated

Simultaneous estimation of individual diet estimates, and a common vector of CCs was proposed by Bromaghin et al. (2017) to potentially avoid the need to carry out onerous CC experimental studies. The diet and CC estimates can be computed using the function p.sim.QFASA, which employs the AIT distance as in Bromaghin et al. (2017). Similarly, “Simultaneous Unified Fatty Acid Signature Analysis” (SMUFASA) was developed by McNichol (2021) and extends the MUFASA approach by maximizing a complex likelihood over a single compositional diet vector and simultaneously a vector of CCs. Individual diet estimates can then be obtained by passing SMUFASA's estimated CCs to any one of the diet estimation methods discussed above. Note that p.SMUFASA, like p.MUFASA, requires numerical integration and a downside to this method is also the associated significant computational burden. At the time of this work, manuscripts detailing the MUFASA and SMUFASA methodology, including a simulation study and real‐life results are undergoing a peer review process. A modeling workflow vignette for p.SMUFASA is also provided with the package.

A summary and corresponding brief description of the diet estimation functions are provided in Table 1 and Table S1 in the Supporting Information.

**TABLE 1:** Diet estimation functions in the QFASA R package.

Function name	Description
p.QFASA	Computes the QFASA diet estimates using either the Aitchison, Kullback–Leibler or chi‐square measure of distance
p.MUFASA	Computes the diet estimates using an MLE approach where the assumed model involves random effects and numerical integration
p.MLE	Computes the diet estimates using an MLE approach where the assumed model uses prey means
backward.elimination	Computes diet estimates by first selecting the “best” prey database using a backward elimination approach and p.MLE
forward.selection	Computes diet estimates by first selecting the “best” prey database using a forward selection approach and p.MLE. A starting subset of prey types is optional but recommended
p.SMUFASA*	Computes calibration coefficient estimates alongside an overall diet estimate using the p.MUFASA approach

p.sim.QFASA*	Computes calibration coefficient estimates alongside individual diet estimates using the p.QFASA approach with Aitchison distance

*Note:* Where * denotes functions that estimate CCs in addition to diet estimates.

### 2.2 Inference

Simultaneous confidence intervals were developed by Stewart and Field (2011) for the true common diet of a group of predators. The intervals are not based on asymptotic normality and can be used when the sample sizes of the predator and prey are less than the number of FAs. The procedure utilizes parametric bootstrapping and yields intervals that need to be shifted by an estimate of bias. The function comp.meth returns the bias‐corrected intervals. Among a variety of parameters whose values are pre‐set, the function requires a matrix of diet estimates (where each row corresponds to a predator) as well as the predator and prey FA signatures. To date, confidence intervals using this technique have only been assessed for diet estimates obtained using p.QFASA.

The function testfordiff.ind.pval returns the *p*‐value corresponding to a multivariate permutation test for a difference in independent samples of FA signatures (Stewart et al. 2014). The test statistic measures the chi‐square distance between the two samples to account for the data being compositional and often with zeros (Stewart 2016). We note that this method was assessed in Stewart et al. (2014) on predator signatures where changes in diet resulted in significant changes in the signatures. It is unknown whether significant differences (as determined by this method) between, for example, prey types translate to more accurate diet estimates as might be expected.

### 2.3 Repeatability

Recently, a measure of repeatability was developed to measure temporal consistency in individual diet estimates (Stewart et al. 2021). The measure is an extension of the estimate of the intraclass correlation coefficient for the univariate two‐way case (see Mcgraw and Wong 1996), where a nonparametric multivariate analysis of variance (MANOVA) with the chi‐square distance is used to manage both the compositional nature of the diet estimates and the zeros. The function comp.rep computes the repeatability (and an associated bootstrapped based confidence interval, if desired) for both the balanced and unbalanced (i.e., with missing data) cases. The measure was found to be biased, so the results returned are bias corrected (Stewart et al. 2021).

### 2.4 Model Assessment

The QFASA R package offers a few simple functions for carrying out procedures that often accompany QFASA research and can be used to assist with model assessment.

A basic requirement for successful diet estimation via FA analysis is that potential prey in the predator's diet must be relatively distinct in their FA signatures across prey types. As such, prior to using the diet estimation techniques, it is recommended that the prey database be evaluated to determine which species may be similar in their FA signatures and, consequently, difficult to estimate. The QFASA R package offers two functions, prey.cluster and prey.on.prey, that can be useful in highlighting potentially problematic species. The function prey.cluster yields a dendrogram plot based on the distance between the average prey type FA signature in the supplied prey database, where users have the option of selecting either the KL, AIT, or CS distance. The function prey.on.prey follows the “leave‐one‐prey‐out” approach published in Bromaghin et al. (2016). With this procedure, each prey in the prey database is treated as a predator and its diet is estimated using p.QFASA without the incorporation of CCs or fat content. For prey types that are easily distinguishable, we would expect QFASA to work well, resulting in the average diet, across individuals of a given prey species, to be near one. Conversely, for confounding or problematic prey types, the diet estimates tend to be much less than one.

Pseudo‐predator generation, originating in Iverson et al. (2004) and now customary for evaluating QFASA methodology, allows researchers to apply their developed methods to samples of pseudo‐predators with known diets, which then allow for a comparison between the computed diet estimates and the true (known) diet of the pseudo‐predators. The traditional approach for creating a pseudo‐predator follows the basic algorithm of choosing a true diet, sampling the prey database (usually with replacement) proportionately, and then forming a linear combination of the true diet and (average) sampled prey type FA signatures. This procedure, which can be carried out using the function pseudo.pred, is referred to in Steeves (2020) as a nonparametric approach since simulated signatures do not rely on any underlying distributional assumptions. Calibration and fat content (inputs to pseudo.pred) may also be added to the final signature, if desired, to better mimic the real‐life setting and expected variability in predator FA signatures. The pseudo.pred function allows for either one randomly selected FA signature per prey type to be used in creating the pseudo predator or, alternatively, the mean signature of each prey type after resampling. For a discussion on generating pseudo‐predators that reflect the true variation in the FA signatures of the predator of interest, see Bromaghin (2015).

Pseudo‐predators may also be generated parametrically using the function pseudo.pred.norm, which assumes that the transformed prey signatures are multivariate normally distributed, allowing for different specified mean vectors for each prey type and a common variance–covariance matrix.

## 3 Results

The QFASA R package contains sample data, which are used in the function examples in the package help files. The sample data include 10 mock seal (predator) FA signatures, a prey database with 11 prey types (sample sizes are shown in Table 3), a vector of CCs, and a list of the desired (39) FAs to be used in the analysis. The predator data, for example, can be accessed through the call data (predatorFAs). We make use of the package's sample data in the examples below. It is important to note that the data in the package are artificial and therefore no inferences or generalizations can be made from the analyses below concerning the prey and the diets of seals, nor the performance of the methods.

### 3.1 Preliminary Analysis

**FIGURE 1:**
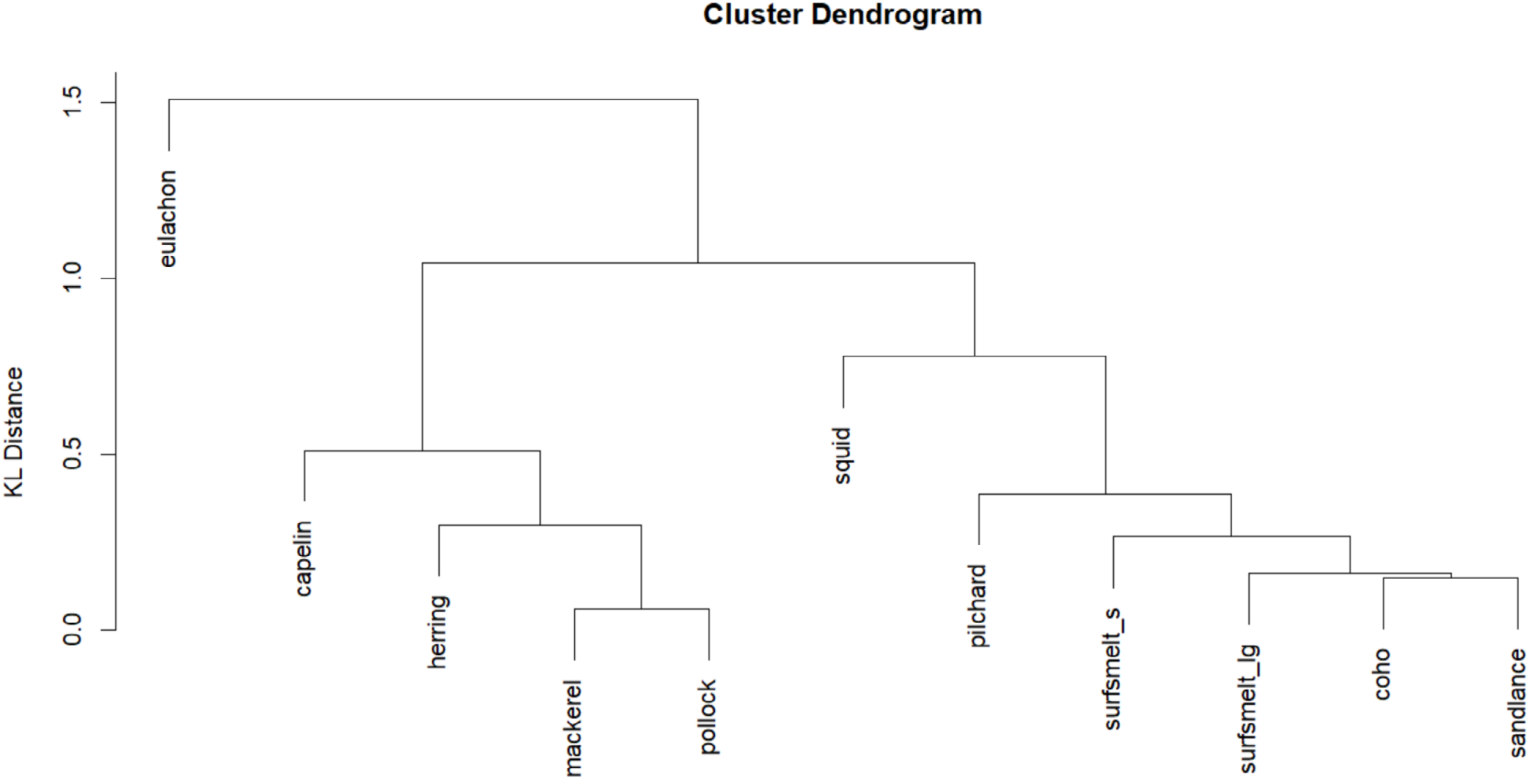
Dendrogram of prey database (preyFAs) in the QFASA R package after extracting the subset of FAs in FAset and using the KL distance.

In addition to the selection of an appropriate subset of FAs and calibration coefficients, the prey database used for diet estimates requires careful consideration. When the potential number of prey types is large, the function forward.selection may be used as a first step in trimming the prey database. This was not necessary for the sample data, as the number of prey types is relatively small and much less than the number of FAs. Once the prey types are selected, it is important to explore the similarity between the prey signatures. Applying the function prey.cluster to the prey database with the FAs in FAset extracted (and signature re‐normalized), the KL distance, and the “average” method of agglomerative hierarchical clustering, we obtain the dendrogram in Figure 1.

Notice that surfmelt has been divided into surfsmelt_l and surfsmelt_s to account for potential differences in the FA signatures of small and large surfsmelt. While the dendrogram suggests only a small degree of separation, this can be investigated further through the prey.on.prey function.

The results of prey.on.prey (using the KL option) are displayed in Table 2, where each row represents the average diet estimate of the corresponding prey type. We would expect prey types with minimal FA signature overlap to have diagonal values close to one. When compared with Figure 1, it is not surprising that euclachon and squid are well estimated (based on their large diagonal values) in Table 2. We note that QFASA does not appear to confuse the two surfsmelt prey types with each other, though surprisingly, squid is appearing in both of their diets in moderately large (13% on average) amounts.

**TABLE 2:** Prey on prey results using KL distance and the sample prey database in the QFASA R package.

Prey type	Average diet estimate per prey type via p.QFASA										
	Capelin	Coho	Eulachon	Herring	Mackerel	Pilchard	Pollock	Sandlance	Squid	Surfsmelt_s	Surfsmelt_l
capelin	**0.9**	0.01	0	0	0	0.01	0	0.01	0.06	0	0.01
coho	0	**0.68**	0.01	0	0.01	0.05	0	0.05	0.05	0.01	0.12
eulachon	0	0	**0.95**	0	0	0	0	0.01	0.01	0	0.02
herring	0.08	0	0.05	**0.66**	0.06	0.08	0.01	0.05	0	0	0.01
mackerel	0.01	0	0.01	0.02	**0.78**	0	0.12	0	0.03	0	0.02
pilchard	0	0.01	0.02	0	0.01	**0.92**	0.01	0.02	0.01	0	0
pollock	0.04	0	0	0.04	0	0.03	**0.84**	0.01	0.01	0	0.02
sandlance	0	0.01	0.02	0.01	0	0.05	0.01	**0.79**	0.07	0.02	0.01
squid	0	0	0	0	0	0.01	0	0	**0.98**	0	0
surfsmelt_lg	0.01	0.02	0.03	0	0.01	0.03	0.02	0.06	0.13	**0.68**	0.02
surfsmelt_s	0	0.03	0.14	0.01	0	0.01	0	0	0.13	0.01	**0.66**

*Note:* When there is minimal overlap in the FA signatures of the prey types, we expect the diagonal elements (highlighted and bolded in the table) to be close to 1.

### 3.2 A Comparison of Diet Estimation Techniques

#### 3.2.1 CCs (Assumed) Known

**FIGURE 2:**
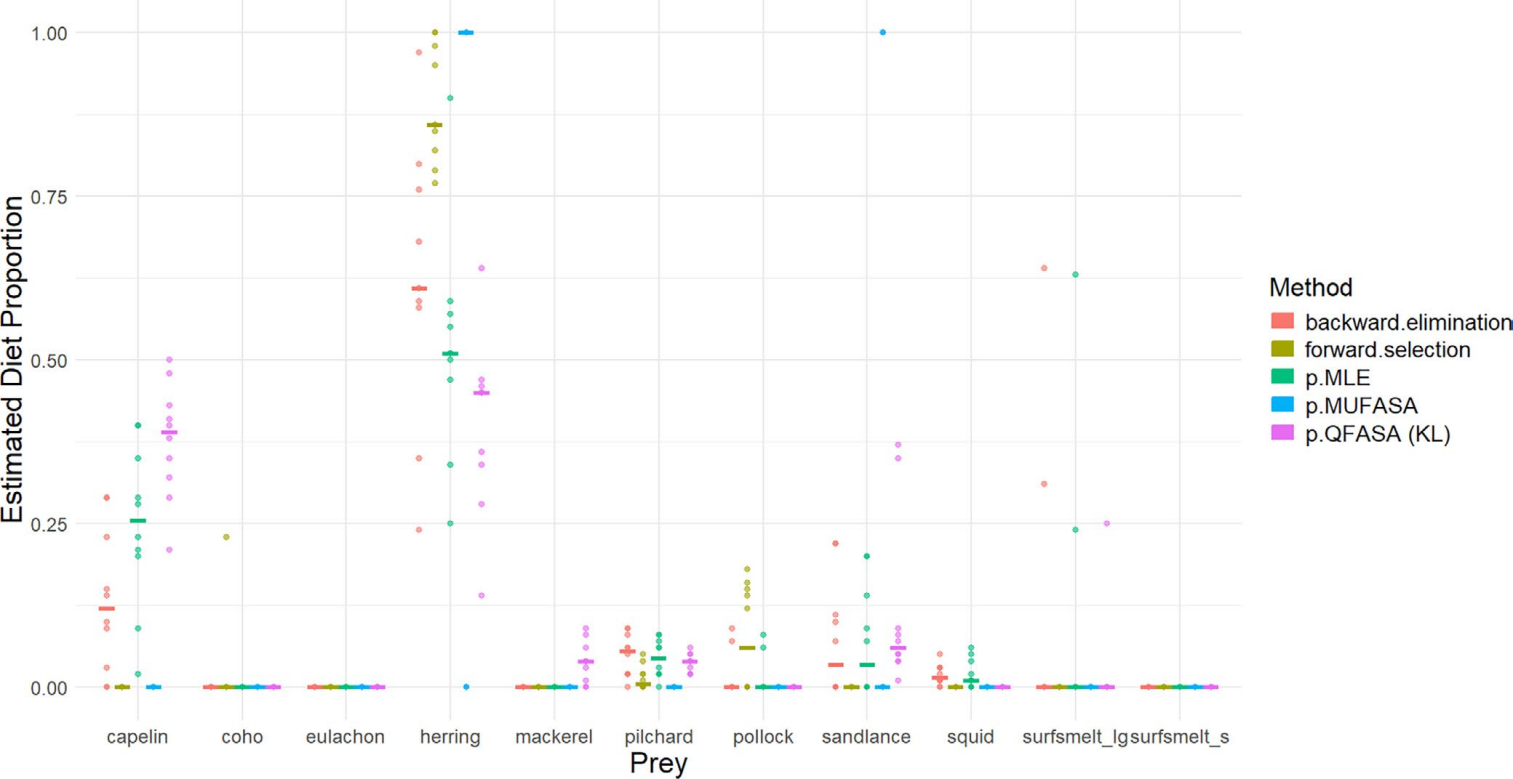
A comparison of the diet estimation techniques applied to the sample data in the QFASA R package that require as input a set of calibration coefficients, namely p.QFASA, p.MUFASA, p.MLE, forward selection, and backward elimination. The solid line represents the median of the diet estimates over the 10 predators.

The results of the QFASA R package diet estimation methods p.QFASA, p.MUFASA, p.MLE, forward selection, and backward elimination, applied to the package sample data, including the sample CCs, are displayed in Figure 2. Code to obtain the diet estimates may be found in the help file of these functions. We note, however, that unlike in the package example for forward selection, we chose not to input starting species.

It is interesting to note the substantial differences in the estimates from the five methods, particularly for the estimates of capelin and herring. While p.QFASA estimates the diets to be predominantly a mix of capelin and herring, p.MUFASA estimates the diet of 6 of the 10 (fake) seals to be solely herring. Note that herring and capelin may be similar in their FA signatures based on Figure 1. The diet estimates returned by p.MLE appear to resemble a mixture of those from p.QFASA and p.MUFASA, which is not surprising since the method itself combines aspects of both of these methods. There are some differences in the estimates produced by the two prey selection methods, which both utilize the likelihood from p.MLE.

Using conf.meth and p.QFASA (with the KL measure of distance), simultaneous 95% confidence intervals for the true diet are given in Table 3.

**TABLE 3:** The 95% simultaneous confidence intervals returned by conf.meth using estimates from p.QFASA with the KL measure of distance.

Prey type	Sample size	95% confidence interval for diet proportion
capelin	54	(0.284, 0.488)
coho	38	(0.000, 0.039)
eulachon	30	(0.000, 0.032)
herring	23	(0.304, 0.550)
mackerel	24	(0.016, 0.159)
pilchard	18	(0.010, 0.098)
pollock	17	(0.000, 0.020)
sandlance	15	(0.042, 0.342)
squid	43	(0.000, 0.035)
surfsmelt_lg	30	(0.000, 0.141)
surfsmelt_s	10	(0.000, 0.028)

#### 3.2.2 CCs Estimated

Here we compare the two existing functions, namely p.sim.QFASA and p.SMUFASA, that simultaneously estimate the diet and CCs given a sample of predator FA signatures. For p.SMUFASA, the final diet estimates are obtained through p.QFASA (KL option) using the estimated CCs.

**FIGURE 3:**
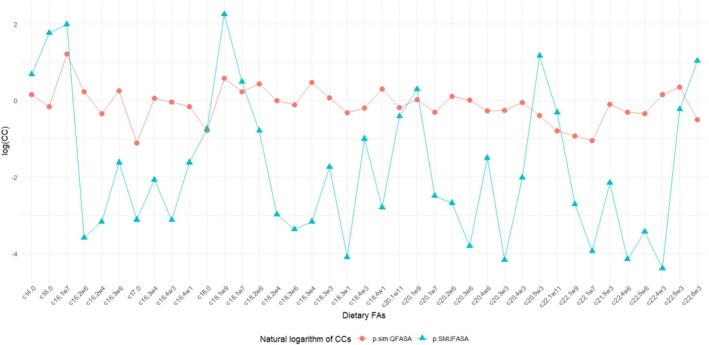
A comparison of the estimated (log‐transformed) CCs obtained using p.sim.QFASA and p.SMUFASA applied to the sample data in the QFASA R package.

**FIGURE 4:**
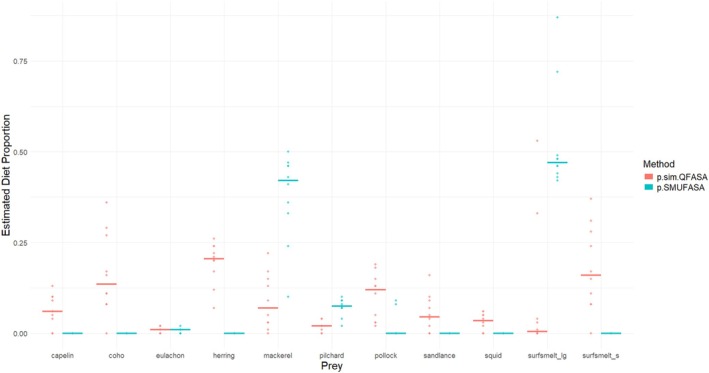
A comparison of the estimated diets from p.sim.QFASA and p.SMUFASA applied to the sample data in the QFASA R package. The solid line represents the median of the diet estimates over the 10 predators.

It is evident from Figure 3 that estimated CCs using the package example data are quite different for most of the FAs on the log‐transformed scale. We note that a log(CC) near zero corresponds to a FA that matches closely in the predator and the prey FA signatures (since in this case the CC is near 1), while a value of log(CC) far away from 0 suggests that the FAs are quite different. It is apparent that these differences resulted in vastly different diet estimates (see Figure 4). The estimated proportions from p.sim.QFASA tend to be relatively small and suggestive of a varied diet, while p.SMUFASA resulted in diets consisting of large amounts of mackerel and sufsmelt_lg, and some pilchard only. Both methods yielded diet estimates that differed from those in Figure 2 where CCs are treated as known.

### 3.3 An Example Using Simulated Data

In this example we apply the function testfordiff.ind.pval to test for a difference in two samples of simulated predator FA signatures, each of size 100, where the function pseudo.pred is used to generate the signatures from the sample prey database in the package. A sample function call is provided in the help file example for pseudo.pred and we simply used a for‐loop to create each of the two samples. We opted to use the default argument of preysize = 2 (for this setting, the mean of resampled prey from each prey type is used in the formation of the pseudo predator) as well as vectors of ones for both the calibration coefficients and fat content. We note that for these choices of parameters, we would not expect the generated signatures to be as variable as those in real‐life data. We examined two cases: for the first case, both samples were generated using Diet 1 in Table 4 and for the second case, one sample was generated with Diet 1 and the other with Diet 2 (Table 4). For case 1, the signatures should not be significantly different and, as expected, testfordiff.ind.pval returned a large *p*‐value (0.408) while for case 2, when the two samples were generated with different diets (Diet 1 and 2), the function returned a *p*‐value of zero.

**TABLE 4:** The diets used in pseudo.pred to generate a sample of pseudo predators.

Prey	Diet 1	Diet 2
capelin	0	0.2
coho	0	0.2
eulachon	0	0.2
herring	0.2	0
mackerel	0	0.2
pilchard	0.2	0
pollock	0	0.2
sandlance	0	0.2
squid	0.2	0
surfsmelt_lg	0.2	0
surfsmelt_s	0.2	0

## 4 Discussion

The QFASA R package encompasses the latest methodology for carrying out diet estimation via FA signature analysis, as well as functions for model assessment and inference. The package is regularly updated to ensure that new research and developments are incorporated. The package is therefore continually evolving, and while at this time some of the methodology has been published, manuscripts on some of the newer procedures are in preparation or have been submitted for review. Nonpublished functions should therefore be used with prudence. We note further that we did not discuss in detail the over 30 package functions, nor the accompanying four vignettes, which are available.

Due to the dimension of the data and the complexity of some of the algorithms, including the use of resampling schemes, some methods are computationally slow. The functions for obtaining confidence intervals for diet (conf.meth) and repeatability (comp.rep with CI = TRUE) require a considerable amount of processing time. The functions p.MUFASA and p.SMUFASA, in particular, are memory intensive. Generally speaking, the larger the number of prey types and/or the number of predators, the more computationally intensive the functions become.

It is worth mentioning that there exist functions in the package qfasar (Bromaghin 2017) that may be used in conjunction with the QFASA R package, particularly in the data preparation stage. For example, while traditionally fatty acid proportions are scaled to sum to 1, prep_sig allows for the FA signature to be left unscaled or augmented with the insertion of another proportion that ensures the signature sums to 1. Using this latter option also requires the computation of an additional CC, and this can be carried out in cc_aug in qfasar (see Bromaghin et al. (2016)). In addition, the pred_beyond_prey is useful in revealing FAs in the predator that, particularly after calibration, fall outside the range of the prey FAs and could potentially bias results (Bromaghin 2017). Once the FA signatures and CCs are prepped, they may be used in the QFASA R package functions after proper formatting and addressing some nuances.

As a further future enhancement, the development of a Shiny app in conjunction with the QFASA R package is underway to facilitate the inputting of data and displaying of results. This will allow inexperienced R users to not only compute the diet estimates more conveniently, but also to explore efficiently the effect of the measures of distance, FA subset, CCs, and prey type choices on the estimates.

## Author Contributions

**Connie Stewart:** conceptualization (lead), methodology (lead), software (equal), writing – original draft (lead), writing – review and editing (lead). **Justin Kamerman:** conceptualization (supporting), software (equal), writing – review and editing (supporting). **Jennifer McNichol:** methodology (supporting), software (supporting), writing – review and editing (supporting). **Holly Steeves:** methodology (supporting), software (supporting), writing – review and editing (supporting). **Tyler Rideout:** methodology (supporting), software (supporting), writing – review and editing (supporting).

## Conflicts of Interest

The authors declare no conflicts of interest.

## Supporting information


Data S1.

## Data Availability

This manuscript uses sample data available in the QFASA R package.
