# Supplementary material for: QFASA: A Comprehensive R Package for Diet Estimation via Fatty Acid Signature Analysis
Source: Ecol Evol. 2025 Mar 12;15(3):e71090. doi: 10.1002/ece3.71090 (PMC11903200; doi:10.1002/ece3.71090)
Supplement: Supplementary file 1 — Data S1. [file ECE3-15-e71090-s001.zip › ece371090-sup-0019-Supplementary Material.docx]

**TABLE 1** Diet estimation functions in the QFASA R package where * denotes functions that estimate CCs in addition to diet estimates.

| **Function Name** | **Description** |
| --- | --- |
| p.QFASA | Computes the QFASA diet estimates using either the Aitchison, Kullback-Leibler or chi-square measure of distance. |
| p.MUFASA | Computes the diet estimates using an MLE approach where the assumed model involves random effects and numerical integration. |
| p.MLE | Computes the diet estimates using an MLE approach where the assumed model uses prey means. |
| backward.elimination | Computes diet estimates by first selecting the “best” prey database using a backward elimination approach and p.MLE. |
| forward.selection | Computes diet estimates by first selecting the “best” prey database using a forward selection approach and p.MLE. A starting subset of prey types is optional but recommended. |
| p.SMUFASA^*^ | Computes calibration coefficient estimates alongside an overall diet estimate using the p.MUFASA approach. |

| p.sim.QFASA^*^ | Computes calibration coefficient estimates alongside individual diet estimates using the p.QFASA approach with Aitchison distance. |
| --- | --- |
